# Reimagining forensic science – The mission of the forensic laboratory

**DOI:** 10.1016/j.fsisyn.2021.100153

**Published:** 2021-06-02

**Authors:** Ray A. Wickenheiser

**Affiliations:** New York State Police Crime Laboratory System, 1200 Washington Avenue, Building 30, Albany, NY, 12226-3000, USA

**Keywords:** Reimagining, Forensic science, Forensic laboratory, Forensic service, Backlog

## Abstract

The year 2020 brought the COVID-19 pandemic, and increased focus on American racial injustice and victims’ rights, spurring a reimagining of law enforcement and justice services. As forensic laboratories serve investigation and justice with objective data to drive investigations, prosecutions, and exonerations, it is worthwhile to also reimagine forensic science service. With comparators of cost and quality relatively fixed to the consumers of forensic service, service in the form of timeliness of turn-around-time is the main competitive measure of effectiveness. A total backlog can be defined as all cases submitted to the forensic laboratory where a report has not yet been issued. Within a total backlog are the in-analysis backlog and the awaiting start of analysis backlog. By eliminating the awaiting analysis backlog, analysis could begin immediately upon submission. This would provide analysis in as short a time as technology permitted, optimizing the value of forensic laboratory service.

The time has come for a reimagining of forensic science service. The year 2020 brought the COVID-19 pandemic and an increased focus on the use of science and objective data to guide governmental and societal programmatic decisions [1]. It also brought a new view of institutional and systemic racial injustice in the United States, stemming in part from the death of George Floyd in Minneapolis [2]. The ‘Me Too” movement has given voice to victims of crime [3,4], who are demanding more timely forensic results, and expanded legislation that includes law enforcement submission of all sexual assault cases to forensic laboratories where a survivor has consented to analysis [5]. Numerous states and jurisdictions including New York have begun a process of reimagining policing services [6]. As forensic laboratories place a pivotal role in serving law enforcement investigations and the justice system, it is therefore a worthwhile effort to examine and reimagine the role of forensic laboratories in this new and changing environment.

Better data provides for better decisions. While science has long been relied upon to provide information to back sound reasoning and informed decisions, uncertainty and the large negative consequences of potential choices have highlighted the importance of establishing a common set of facts to anchor debate and decision making. The importance of the application of science to improve lives has been further elevated in the public eye with the COVID-19 pandemic. Statistics including the number of new infections, testing rates, positivity rates, hospitalizations, and deaths, have not only been watched by policy makers, but members of the population with increased availability and scrutiny. Sites such as the Johns Hopkins COVID Tracker [7] and New York COVID tracker [8] have increased transparency and participation of the public in judging policy, but also in determining their own behavior and its impact on their and their family's safety. Reduced timelines for development of scientific products, including vaccines and antiviral treatments such as monoclonal antibodies, have been critical in saving lives [9]. The push towards shortening delivery timelines has further increased the importance of proving data to support decisions, particularly for vaccine and COVID treatments [10]. Analytical lab capacity needed to be built to deliver the volume, accuracy, and timeliness necessary to meet demand [11]. The critical nature of providing timely and accurate data has been demonstrated now more than ever. Response time matters.

The data backing forensic laboratory analyses are objective, and aside from the rare exception of DNA phenotyping in unsolved cases, it does not see potential ancestry nor place bias against a particular group. Investigations guided by objective analytical results are a part of the answer to combat systemic racism, where results can be verified or refuted through review of data, or retesting if need be. Victims of crime need not be victims of the justice system through non-testing of their cases. Science guides justice. Objective analytical forensic data protects the crime victim and wrongfully accused alike.

In many ways, these developments felt through the trials of the pandemic response and social unrest emphasize the critical role forensic laboratories play in investigating crime. Forensic laboratories parallel medical health laboratories in providing actionable data [12]. Despite the changes to society brought about by the pandemic, crime did not take a break, with demographics shifting from decreased robberies and property crimes, to increased drug overdoses, domestic disputes and shootings [13,14]. Similar to medical laboratories, forensic laboratories provide independent, objective data, which is based upon scientific principle and hence reproducible. Samples are typically not consumed, so that testing can be reproduced if necessary. Quality controls to ensure validity of results are included. Accreditation ensures that quality systems are in place to support quality of data and interpretations of results. Documentation of procedures and testing enables discovery for court proceedings. Just as better diagnosis is supported with medical testing, so too are better investigations supported with forensic laboratory analysis. As science guides justice with fact, it is part of the solution. Decisions must be made on sound information, based upon sound data. An investigation with a lab report is better than one without.

To best determine where forensic science can make its largest impact, the reimagining of the field can place it within our new context. In New York State, the state government required local police departments to reimagine their services, by including all stakeholders and interested individuals in the discussion and submit a plan for what policing would look like in their communities. Those without a plan would forfeit state government funding [6]. The process of reimagining essentially looks at a service with a blank sheet of paper, determining what service should be, rather than looking at what it currently is and making modifications.

Hence, to reimagine a forensic science service, one starts at the beginning with what forensic laboratories produce, akin to a business offering. Forensic laboratories have three primary products. The first is testimony, second the laboratory report and third the documentation and data which support the laboratory report and testimony. While the later product category of documentation and data contains a great many items, for simplicity they are grouped together as they share one purpose. That purpose is to provide the supporting data and documentation to back the interpretations found in the laboratory report and any potential testimony based on that report. Without that supporting data and documentation, the laboratory report would not withstand scrutiny, nor be able to be produced in the first place. This support is much like a pyramid, with testimony at the top, being based upon the report and the data lying beneath (see Fig. 1, the forensic laboratory product pyramid).

**Fig. 1:**
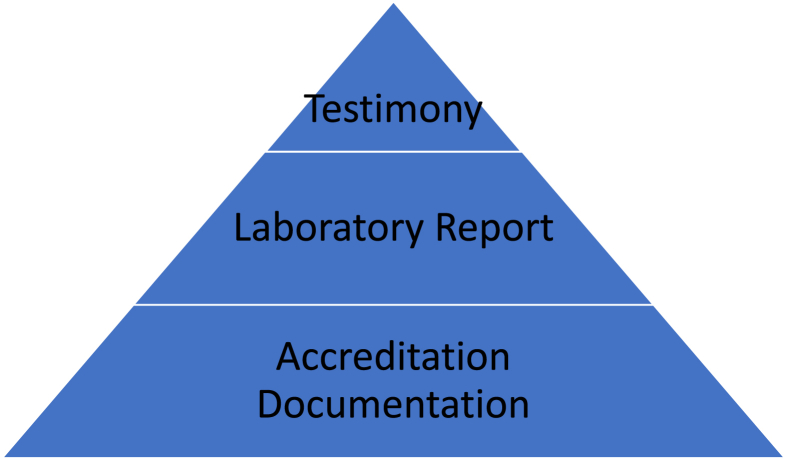
The forensic laboratory product pyramid.

The laboratory report typically contains three types of general product categories. These are identification, comparison, and investigative leads. Identification refers to the identification of an unknown item or substance, such as produced in Drug or Toxicology Sections when identifying and quantifying a controlled dangerous substance. Comparison includes comparing an item or substance to a known item, such as comparing a fingerprint or DNA from a crime scene to a suspect. Investigative leads typically involve comparing evidence found at a crime scene, such as an expended cartridge casing, to a known database, to provide investigative leads previously unknown to investigators, as is provided via NIBIN (National Integrated Ballistics Information Network). All of these examination types add value to crime scene evidence and investigations. However, notably as investigative leads provide new suspect information to investigators, their timeliness is of particular importance.

Businesses are measured by their clients and thereby compete on three dimensions, these being price, service, and quality. The vast majority of forensic laboratories are run by government entities funded by taxpayer dollars, hence price is often removed from the equation, as very few forensic laboratories charge a fee for service. Quality demanded by the justice system is also not variable, as substandard forensic testing is deemed unacceptable, particularly in accredited forensic laboratories. Without price and quality as selective processes, service is therefore the variable that is the measure of a forensic laboratory. Forensic service is measured in terms of turn-around-time, which is how quickly analysis is provided via a laboratory report. While most forensic laboratories triage cases to respond more quickly to more urgent cases, a backlog of uncompleted cases significantly hampers response time.

Law enforcement entities submitting cases do not typically have a choice of which forensic laboratory they submit their cases for analysis. The catchment areas for cases are relatively fixed, as law enforcement jurisdictions are generally stable, with forensic laboratories historically in place to serve specific agencies. The target market is predetermined, with a set group of consumers assigned to each forensic laboratory. As a result, the choice is not where to go for forensic analysis, but rather which cases and items to submit. Therefore, investigators become their own triage of cases and items when timeliness of service is not meeting their need. The result becomes pent up demand for forensic analysis, or rather a build-up of analyses that are not conducted as cases and items are self-edited through non-submission. An investigator is less likely to submit a case or item when more important case analyses are not yet complete. This logic of pent up forensic demand is backed by the findings of a negative elasticity of demand of -1.29 by Dr. Paul Speaker, which means that for every case the backlog is reduced, 1.29 new cases are submitted to the lab [15]. Furthermore, those new cases also include a greater number of items.

While investigator self-triage of cases represents a danger to cases not being supported with best evidence, it also represents an opportunity. Training and communication with investigators by forensically experienced multidisciplinary staff can serve to make informed choices as to which cases and items within those cases would most benefit from forensic analyses. Crime scene investigators typically over collect evidentiary items as they have one opportunity to process the crime scene, when full circumstances surrounding the crime are not known. As these circumstances come to light, selection of probative items will yield a subset of seized items whose analyses will direct further potential analyses. With positive forensic findings, further analyses are often negated. This layered or tiered approach is better supported by incorporating forensic experts and their expertise with crime scene investigators in a partnership. COVID-19 has fast forwarded remote working capability, which can be applied to this partnership via a virtual forensic scientist as part of a proactive crime scene response team. This partnership enables improved evidence recognition, collection, and expedited selection of probative items for forensic analysis.

Primary customers of the forensic laboratory are investigators and prosecuting attorneys. Investigators are looking to build support for their case, which if charges are substantiated, they pass on to prosecuting attorneys. In most instances, both entities work very closely together. Prior to a suspect being developed, which occurs in no-suspect cases pursuing forensically developed investigative leads, it is the investigator who is the main client. Crime survivors, defendants and defense attorneys are also stakeholders; however, case submission and investigative choices do not often involve them. They are motivated by many of the same factors with respect to forensic science, however. Defendants and their legal representation care about discovery, transparency, objectivity, and lack of bias. While these things also matter to crime survivors, they have a particular interest in transparency as it relates to response time and notification of their case outcome, provided it does not hinder the case investigation. Crime survivors are ensuring their voices are heard through crime survivors’ rights organizations, who advocate for appropriate tracking and notification throughout the investigative, analytical, and judicial process [16].

The inclusion of stakeholder representatives in a Forensic Laboratory User Group can provide critical direction to forensic initiatives. To make best use of stakeholder time, a forensic laboratory must provide education on its programs, and provide an options analysis when specific input on decisions impacting the stakeholders are considered. Including stakeholders improves decision making, avoids potential costly mistakes and creates improved communication and delivery of service through selection and implementation of the best option.

Many forensic laboratory leaders are frequently not in a position to request additional resources for programs and are left to operate within existing resources. Education of decision makers and stakeholders at a level above or outside of forensic laboratory leadership is necessary to allocate resources on a wider basis to optimize the investigative and justice system as a whole, rather than focusing specifically upon forensic laboratories in a vacuum. Requesting forensic resources to battle backlogs is not new. Reimagining the investigative and justice system including forensic laboratories is.

Put simply, the mission of forensic laboratories is to maximize the value of evidence [17]. Providing maximum value of evidence through applied science occurs through several mechanisms, including non-destructive flow of analysis, sensitivity, specificity, and timeliness. While it can be debated these features have aspects that fit both the quality and service aspect of competition, it is inarguable that a forensic laboratory that can provide more discriminating, conclusive and timely evidence analysis is providing a better service.

Non-destructive flow of analysis means that the sample is not consumed, as in some of the sample is preserved for different analysis types so all of the data can be utilized. The process flow of analyses in forensic laboratories should be tailored to conduct the most probative and least destructive analyses first, with the least discriminating, most sample consuming and destructive analyses last, so as to wring out the most information from each available sample.

A practical case example from the author's experience demonstrates the application of non-destructive flow of analysis. In this case, a male homicide suspect with wet bloody hands rifled through business cards the victim gathered moments earlier while she searched for jobs nearby. Deposited on a recently obtained business card was one of the suspect's fingerprints, made with the blood of the homicide victim. Maximizing the value of the evidence through non-destructive flow of analysis dictated the Forensic DNA Analyst worked hand in hand with the Latent Print Analyst to obtain a sample of blood which was a component of the fingerprint, yet would not hinder the fingerprint comparison. The Latent Print Analyst documented the evidence photographically and directed the Forensic DNA Analyst to sample the least vulnerable area of the print for bodily fluids. As a result, analysis provided a full DNA profile which matched the victim and a latent fingerprint that matched the suspect. This combination presented compelling evidence of the details of the crime, effectively placing him at the crime scene with hands wet with the victim's blood, going through her personal belongings. The suspect was subsequently found guilty of the homicide.

Sensitivity in the context of forensic science refers to the capability to generate a reliable result with a small amount of material. As discussed for non-destructive analyses, providing a result with a smaller amount of material increases the number of cases that can successfully analyzed. Optimizing sensitivity is a moving target, as there will always be cases with sample sizes too small to reliably analyze. However, improved sensitivity will increase the number of samples and cases that are successful, while not consuming excess sample where one analysis may inherently compromise another. Increased sensitivity improves the non-destructive flow of analysis and thereby increases the value of evidence.

Specificity in forensic science refers to the ability to identify a particular substance or source with a high degree of accuracy. To provide a high level of confidence or weight of evidence means that the result is more discriminating, hence the finder of fact is able to rely more heavily on the results in making their determination of fact. While some analyses may not be very specific, evidentiary value may be added never-the-less, so long as the limitations of the finding are clearly stated. However, the more specific the finding, the more valuable the evidence.

Striving for the best in non-destructive case flow, sensitivity and specificity is largely a function of applying best practices and technology. There are approximately 409 forensic laboratories in the United States, existing at municipal, county, state, and federal levels [18]. Many of these forensic laboratories manage backlogs with median turn-around times in excess of 30 days [19]. As a result, laboratories frequently focus on casework analysis and have limited time to conduct evaluation, research, validation, and implementation of new technology. As US forensic laboratories are independently operated, other than loose collaborative efforts, there is little to guide them in terms of adopting new technology outside of striving for continuous improvement. Even being in the top 10^th^ percentile of US forensic laboratories would still mean there are 40 other US laboratories achieving at the same level. One goal of a laboratory is to bring the best of the world of forensic science to their home jurisdiction. Collaborative validation has been demonstrated as a cost saving opportunity to speed the uptake of forensic technology [20]. Therefore, collaboration is not only a necessity, but begs the case of a greater level of coordination of forensic best practices and technology sharing.

Academia, forensic laboratories and suppliers of forensic instruments and consumables have a vested interest in working cooperatively to bring improved technology to bear on forensic cases more quickly. Laboring under case backlogs limits time forensic laboratories have to dedicate to evaluating, validating, and implementing new technology. Academic institutions possess not only scientific expertise, but students requiring projects to complete their studies. While conducing forensic casework in accredited forensic laboratories is not permissible due to accreditation requirements, assisting in validation experiments is. Students not only increase their own market value and experience by furthering forensic science through relevant forensic projects, they gain invaluable forensic laboratory contacts as well. Venders of forensic instruments and consumables are frequently delayed in having their products implemented into forensic casework, due to the large burden of validation. Adding their expertise to that of forensic laboratory scientists and academics further guides validation through their collective experience, shortening validation and implementation times [20].

There is a tremendous opportunity for greater focus of forensic laboratories on macro (multi-case) rather than micro (single case) analysis. Forensic laboratories typically receive submissions from many contributing agencies. Each of these case submissions receive reports, typically with copies only going back to those specific submitters and/or their designees, same agency colleagues and partner prosecuting agencies. Forensic laboratories are aggregators of data, as they retain records of analytical data across their entire catchment area. This data can be aggregated on a macro scale for individual contributing agencies as well as across entire jurisdictions. There exists an excellent opportunity to combine and provide analysis of this larger set of data, including maps of results to demonstrate trends, link cases and facilitate communication between disciplines and jurisdictions [21]. Perpetrators of crime frequently commit multiple crimes types in more than one jurisdiction, hence a fingerprint match in one, a ballistics match in another and a DNA match in a third case be overlayed to provide greater investigative value. Maps of locations of types of offenses, such as controlled substance types via Drug and Toxicology analysis, can provide invaluable near real time data on drug and overdose trends, coupled with the identification of suspects committing crimes.

Shifting lab focus from micro to include macro enables investigating agencies to employ increased community-based policy, responding with crime prevention to problematic areas rather than limited to specific crimes (micro). Early trend recognition, information sharing, and early suspect development further support investigative agency intelligence with actionable macro data. Combining case results between forensic laboratories on a state-wide and national level creates even greater opportunity, such as the National Forensic Laboratory Information System (NFLIS) Drug and Toxicology data compilation and report initiatives [22]. LIMS (Laboratory Information Management Systems) are employed by the vast majority of forensic laboratories, with analysis data being collected in real time. This real time data can be utilized electronically through notification to investigative agencies as results are available, eliminating the lag of traditional mail. Electronic records can facilitate discovery processes for the justice system processes. While monthly, biannual, and annual reports are beneficial, real time reporting of data permits timely and proactive crime prevention strategies.

Timeliness will be a main focus of this discussion as a great number of forensic laboratories have a backlog of cases, which means that investigators and justice system officials are waiting for analytical results. The concept of backlog is frequently not well understood by those outside the forensic community, perpetuated by a variety of definitions and descriptions, including compensation for some number of cases where analysis has begun but is not yet completed. The total backlog is the number of cases that have been submitted to the forensic laboratory, but results have not yet been completed, as in a laboratory report has not yet been issued. There are two separate general categories of cases within the total backlog, namely those cases where analysis has been started but not yet completed (in-analysis backlog) and those cases that are sitting awaiting analysis to begin (awaiting analysis backlog). Regardless of the level of resources afforded a forensic laboratory, there will always be some number of cases in the process of analysis. While a laboratory can shorten the internal flow to reduce the in-analysis backlog, there will always be some number of cases being actively worked upon.

The second backlog category, the backlog of cases awaiting analysis, is frequently a symptom of much greater issues often beyond the immediate control of the forensic laboratory, namely scarcity or inadequacy of resources. Backlogged cases awaiting analysis indicate an imbalance of incoming cases or the demand for forensic analysis, versus the supply of forensic analysis. This situation is well studied and illustrated as the classic supply and demand of economics [23]. In the field of forensics however, unlike standard capitalist economics, increasing demand for analysis does not increase price and thereby entice greater supply to enter into the marketplace. As discussed and established previously, price and quality are not discriminating competitive factors for forensic laboratories. Forensic laboratories typically do not charge submitting agencies, nor can quality vary widely. Therefore, without additional resources, the increase in supply of forensic analysis is limited to increases in forensic laboratory efficiency. Application of best practices can only go so far to increase output with fixed resources. As increasing efficiency has its limitations and improving technology to increase productivity is costly to acquire and implement, supply is thereby relatively tethered to resources. Therefore, the immediate answer to eliminating a backlog of cases awaiting analysis is to increase resources corresponding to the additional supply required to meet demand for analysis.

Forensic evidence located at a crime scene is perishable. That is, it degrades over time, even in the most optimal storage conditions. The best quality of analysis is one conducted immediately. The best value of evidence is also analytical results which are available to customers immediately, as investigative resources are being spent while investigators are awaiting laboratory results, frequently following up on some leads that could be negated by a probative result. The investigative cost curve credited to Gary H. McLeod former laboratory director of RCMP Forensic Laboratory Regina, Canada, demonstrated the highest investigative cost is spent closest to the commission of the crime, with costs tapering off as time progressed [24]. See in Fig. 2 the investigative cost curve of a major homicide investigation which occurred from October 1997 to May 1998 when the case was effectively solved and concluded with the arrest of a suspect. With a response time of 6 months from the submission of the case to the forensic laboratory, there were approximately 1 million dollars in potential operational savings had laboratory results been provided within 30 days. A lead today is better than a lead tomorrow.

**Fig. 2:**
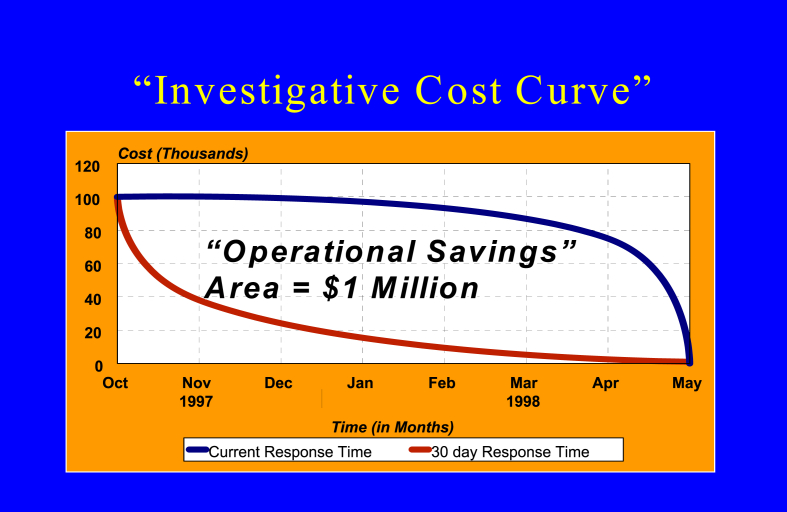
The investigative cost curve. Source: G.H. MacLeod, lab manager (retired), Royal Canadian Mounted Police Forensic Laboratory, Regina, Saskatchewan, Canada [24].

Forensic leads have a time value, which is heavily weighted closer to the commission of the crime. Objective data driven investigation maximizes valuable investigator time. Besides the expense of wasted investigative efforts without results, there is increased opportunity for a perpetrator to commit additional crimes when crimes go unsolved. Crime is frequently committed by recidivist perpetrators; whose crimes frequently increase in frequency and seriousness as their criminal careers progress [25]. Investigations which are not focused on the correct suspect risk the opportunity of wrongfully convicting an innocent non-perpetrator. It is clearly better to eliminate an innocent suspect immediately than to exonerate a wrongfully convicted individual. Unsolved investigations represent delayed justice for crime survivors and their family. While the damage of a crime cannot be undone, solving the crime permits some opportunity for justice and closure for survivors.

In the reimagining process as discussed thus far, the target market of consumers in forensics is fixed, the service offerings relatively predefined, and as a result the comparator of competitiveness has been reduced to timeliness. Therefore, if one was to start at the beginning, the primary question posed is “what level of response time to choose?” The next step is to perform an options analysis to develop and assess choices. Options should be laid out so the best option choice should be obvious to all, being supported by objective metrics and data. The first option choice is to do nothing or accept the status quo. Next, options are developed to cover a range that encapsulates the optimal solution to the question of response time. Finally, depending on cost and other metrics, the decision maker can objectively compare the cost and benefit of choices to arrive at a solution befitting their budget and level of need and solution of that need.

Metrics can be utilized to assist in the assessment and evaluation of choices provided in the options analysis. Public safety impacts all citizens and is a measure of all law enforcement and justice system programs. Early apprehension and rehabilitation provide opportunities to correct rather than punish, as it is easier to build resilient adolescents than repair broken adults. Reduction of wrongful convictions is another measure of using objective data to implicate or exonerate. Victim and family rights, and right to privacy should all be included as measures to weigh various options. Cost is typically an overriding factor, however should include more than the cost of the forensic service itself, but rather total cost to jurisdictions. These cost centers include other impacted departments such as law enforcement, the justice system, social services, incarceration, and cost to society in terms of the damages of crime.

An informal survey was conducted with six different criminal investigative departments regarding an ideal analytical response time for their submitted cases. Their answers were universal. Provide as fast a response as technology permitted. This is not surprising, as forensic laboratories’ work has evolved from identifying items and comparing to known samples, to including databases searches to develop investigative leads. Where identification of a controlled dangerous substance or toxicology was at issue, frequently charges were pending confirmation of suspicions. Plea bargains were more beneficial to the defense when lab results were not in hand. Cases awaiting an analytical result bearing an investigative lead were even more dependent on a quicker response. Whether a comparison is being done to include or eliminate a suspect, or generate a lead in a no-suspect case, the resulting primary investigative information frequently changes the course of an investigation, directing investigative resources away from incorrect suspects towards those more likely committing the crime.

Crimes committed by Paul Bernardo revealed a number of shortcomings in the investigative and forensic system in Ontario, Canada. Due to a large awaiting analysis backlog, Paul Bernardo's known sample sat unanalyzed in a forensic laboratory while he continued to commit escalating crimes. Bernardo's crimes exhibited classic recidivist tendencies. “Between May of 1987 and December of 1992, Paul Bernardo raped or sexually assaulted at least eighteen women in Scarborough, Peel, and St. Catharines and killed three women in St. Catharines and Burlington.” [26] Issues with the delayed investigative and forensic response prompted an inquiry looking for improvements based on that learning experience. In June 1996, Justice Campbell concluded in his report that “A reasonable turnaround time for DNA testing is required, in the range of 30 days.” [26] Additional recommendations in that report included “A continuing commitment of resources is required to achieve and maintain this turnaround time in the face of technological change and rising workload” and “A system is required to better co-ordinate the work of forensic scientists and police investigators” [26].

The Canadian Campbell report is not the only independent examination of backlogs and appropriate response times for the forensic analysis of major crimes to provide reasonable target response times to generate a forensic report of analysis. The National Best Practices for Sexual Assaults: A Multidiscipline Approach also indicates a target response time of 30 days or less for processing sexual assault cases [27]. Likewise, the Report to Congress: Needs Assessment of Forensic Laboratories prepared by NIJ and the Forensic Laboratory Needs Technical Working group provides reference to “the average 30-day backlog is growing faster than the growth in case submissions; the average backlog across all areas of forensic science has grown nearly 250% over the past six-year time period from 2011 to 2017.” [28] Use of a 30-day backlog statistic acknowledges the concept of in-analysis backlog and cases awaiting analysis backlog.

The 30-day response time referenced in the Investigative Cost Curve is based on a relatively achievable forensic response where analysis and interpretation began shortly after submission to the forensic laboratory. This response time assumes that the backlog is in-analysis time, and that there is not a significant backlog of cases awaiting analysis. Notably, the homicide case and DNA technology referenced in the Investigative Cost Curve with a potential 30-day turn-around-time occurred over 24 years ago. Therefore, multiple independent sources indicate 30 days is a responsible and achievable goal for forensic analytical response time.

While the 30-day turn-around time for forensic analysis has been cited and used as a backlog descriptor, there is little justification today for the exact period of 30 days. It is a round number, approximately one month and a relatively achievable goal with existing technology. Similar to the Investigative Cost Curve concept, Justice Archie Campbell's report was delivered in Ontario over 24 years ago. Forensic technology has increased significantly in this time period, such that this goal for analysis and interpretation time is modest, again with the assumption the case is started immediately, worked on continuously and free of major complications. Yet few if any forensic laboratories in the United States can boast a 30-day response time for DNA case submissions and many other analyses [19]. Time a case sits in line awaiting its turn must be eliminated to meet and exceed this 30-day goal. The time is now for reimagining not only forensic science, but the mission of its most basic indicator, turn-around time. Reducing turn-around time to equal the time for actual analysis will provide the maximum value of evidence.

## 1 Conclusion

An imbalance of supply and demand is the root cause of the awaiting analysis backlog. Starting a case upon submission eliminates the time a case awaits analysis. Output (supply) of a good or service must be equal to or greater than submissions (demand) for that good or service to maintain a zero-wait time for analysis commencement. Increasing collaboration and use of best practices is within the forensic laboratories control, hence should be implemented to improve non-destructive analysis flow, specificity, and sensitivity within existing technology. Through conducting only the most probative analyses on the most probative items to restrict the number of analyses per case, capacity can be increased using current resources. If adequate capacity cannot be developed through maximizing internal efficiencies, cases awaiting analysis backlogs will continue to exist indefinitely. Stretching existing resources can only go so far. Beyond this point, additional resources must be tailored to furnish supply to meet demand.

One must question whether forensic analysis, upon which efficient investigation and justice rely, should be rationed? When one locates a bottleneck, which prevents others from achieving greater success, that bottleneck should be examined for elimination. A more typical response would be to evaluate the costs versus the benefits of achieving the goal of starting a case immediately upon submission. Efficiency of investigations and hence public safety is better served by a quicker forensic analytical response. Cases will be investigated whether forensic science technology is applied or not. Without application of economies of scale to eliminate the bottleneck of rare resources, less optimal solutions will be utilized. Investigative resources can be better targeted with improved allocation of resources. Therefore, rather than rationing the bottleneck, efficiency and effectiveness of the larger investigative and justice system dictate it should be removed.

Applying the best tools of technology to solve any problem, be it the COVID-19 pandemic, crime, or systemic inequality is an earmark of our advancing civilization. Via earlier intervention in a recidivist criminal's career, potential savings include investigative resources and improved public safety through less opportunity to reoffend and increased intervention and rehabilitation potential. In conclusion, commencing analysis immediately upon submission keeps the awaiting analysis backlog near zero, resulting in the most optimal backlog solution. Starting analysis upon submission maximizes the value of evidence, and therefore should be the goal of forensic laboratories. Allocating and implementing adequate resources to create analytical supply to meet the demand for analysis is the key to achieving that goal.

## Declaration of competing interest

The authors declare that they have no known competing financial interests or personal relationships that could have appeared to influence the work reported in this paper.
